# Unexpected Populations for Deep Vein Thrombosis: Presentation in an Endurance Athlete

**DOI:** 10.7759/cureus.17495

**Published:** 2021-08-27

**Authors:** Megan LeBlanc, Trenton Cooper, Pankaj Chopra

**Affiliations:** 1 Research, A.T. Still University, Kirksville College of Osteopathic Medicine, Phoenix, USA; 2 Family Medicine, Mercy Gilbert Medical Center, Gilbert, USA

**Keywords:** deep vein thrombosis (dvt), female athlete, thrombosis, venous thromboembolism, unprovoked venous thromboembolism

## Abstract

This case presents an athletic 40-year-old female marathon runner who presented with a headache secondary to dural venous sinus thrombosis and right calf deep vein thrombosis (DVT). Though this is outside of the typical image we portray of a common DVT patient, athletes too experience hypercoagulable risk factors and medical issues, just as their less in-shape peers. This patient’s history of oral contraceptive use, Lynch syndrome, colon cancer, and pregnancy indicates potential risk factors for DVT. Even without these though, it is important to note that every endurance athlete experiences hemoconcentration, dehydration, and inflammation during exercise, training, and competition events. This case demonstrates the need for an increased index of suspicion in endurance athletes. The case exemplifies an all-too-frequent occurrence of allowing our differential to be skewed away from potentially life-threatening conditions like DVT, and their thrombotic sequelae, because of the “textbook population” descriptions of a given disease state. We hope this case will shed needed light on the phenomenon and lead to more controlled research on the probability and pathophysiology for thrombotic events in this broadened population so that its incidence and prevalence in endurance athletes can be accurately reported in the literature.

## Introduction

Venous thrombosis is a common illness that countless individuals suffer from every year, and most health care professionals are able to spot the common population with risk factors that may develop venous thromboembolism (VTE). However, a small subset of individuals are routinely overlooked, as these individuals do not fit the stereotypical mold of sick, recent surgery, and/or bedridden. There are many presentations of VTEs; a few are commonly known as deep vein thrombosis (DVT) and pulmonary embolism (PE) and are generally associated with inactivity; however, marathon runners and other athletes have been seen to develop DVTs as well, which goes against our preconceived notion of the general DVT population. These athletes may be potentially exposed to thrombogenic risk factors while training and performing, such as injury, inflammation, dehydration, hemoconcentration, and contraceptive use [1].

VTEs are said to occur with an incidence of 1/1000 individuals annually in adult populations before 40 years of age. Two-thirds of these cases are DVTs alone while 1/3 are PEs with or without preceding DVTs associated [2]. The incidence only increases as we age with 5-6/1000 individuals annually by 85 and 7/1000 above this age [3-4]. The highest risk of VTE occurs in African Americans, followed by Caucasians, Hispanics, and Asian/Pacific Islanders [5]. There is unfortunately not currently established data for incidence in the athlete population subset specifically, which may be due to the rarity, issues with diagnoses/clinical suspicions, or even that some VTEs undergo self-resolution before they are found. There are numerous factors that contribute to the development of DVTs, but the general consensus is that patients have an underlying medical condition or have been immobilized for a period of time. Additionally, the most common complications that result from DVTs are pulmonary embolisms, chronic venous insufficiency, and post-thrombotic syndrome.

Diagnosis of these events are dependent on pre-test probability and whether or not patients have risk factors that could contribute to VTEs. Common risk factors are recent surgery, immobility, obesity, family history of VTE, and oral contraceptive use, to name a few. Commonly, the Wells Score is used to categorize individuals into groups based on different criteria into high, moderate, and low pretest probability of having a DVT [6]. Once the pre-test probability is determined, a D-dimer assay is obtained [7]. Due to the high sensitivity but low specificity, this assay is able to essentially rule out a VTE, however, if positive, an ultrasound needs to be conducted [8]. If a DVT is present, the ultrasound will show non-compressible obstruction in the venous system of the lower extremity.

Screening athletes for DVTs is currently not a common practice, however, due to potential exposure to thrombogenic risk factors, clinicians need to keep this disease in mind when interacting with the said population due to the morbidity a VTE can cause. In this case, we give an example of a healthy female athlete who is a marathon runner and subsequently developed a VTE.

## Case presentation

**Figure 1:**
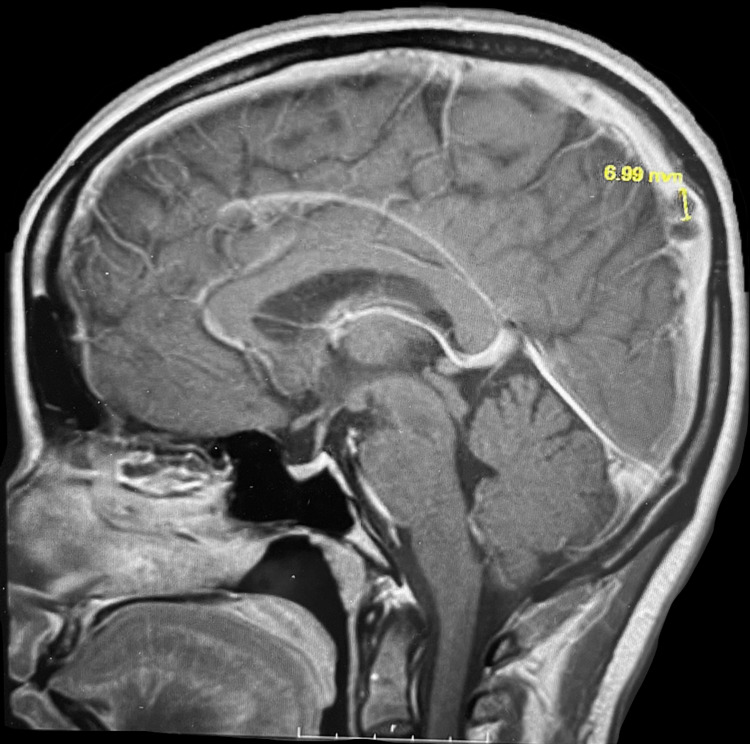
MRI of Brain With a Sagittal Sinus Filling Defect

**Figure 2:**
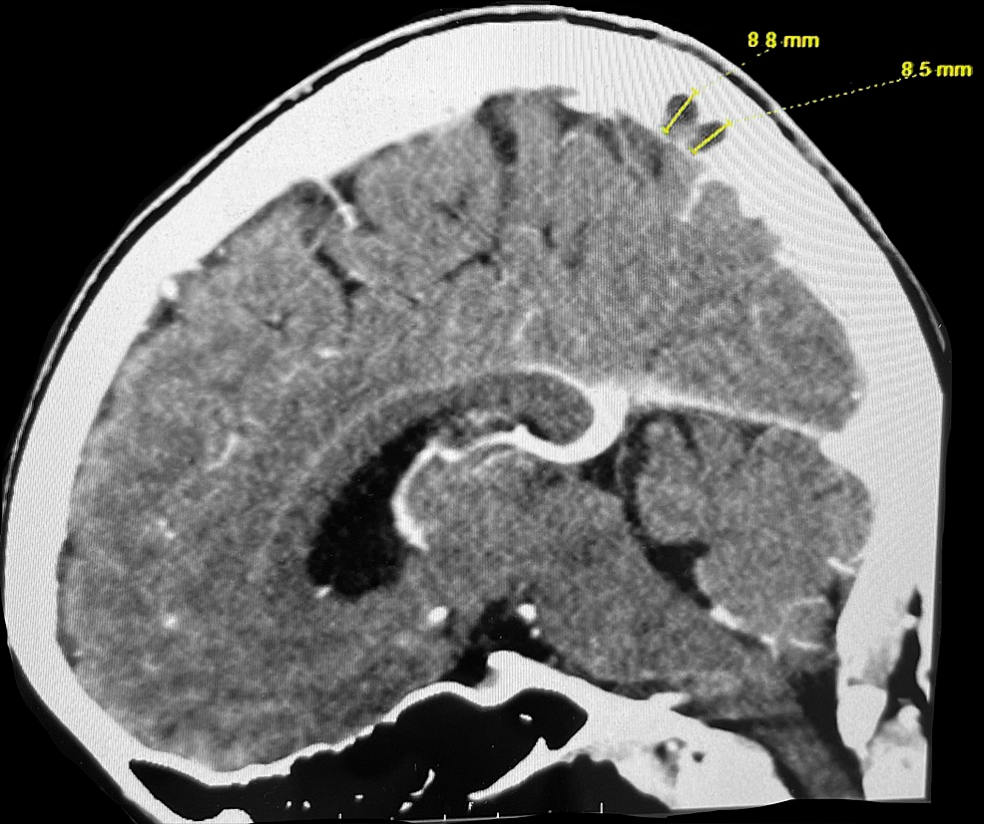
CT Venogram With Sagittal Sinus Filling Defects

This case reports on an athletic 40-year-old Asian female who presented with the chief complaint of left-sided headache with radiation to the left eye, jaw, and shoulder for two consecutive days. Importantly, approximately two weeks prior to hospitalization and symptomatic headache, she completed a full-length marathon without any problem or incident. She had trained for this event beforehand and exercises regularly. She is a 5’3”, 49.75 kg athlete with a body mass index (BMI) of 19.43 kg/m2. The patient reports moderate severity of her headache, describes the pain as significant pressure and reports that Tylenol, Advil, and Midol were all ineffective for symptom relief. Though Advil typically relieves any headaches she has experienced prior to the current incident, none of the over-the-counter interventions proved to be effective for this event. She denies cognitive deficit, language disturbances, problems with vision, or previous history of migraines. Neurological examination demonstrated cranial nerve 2-12 fully intact, sharp discs bilaterally on fundoscopic examination, and equal 5/5 motor strength on bilateral upper and lower extremities for both proximal and distal flexion, extension, and grip strength. There is no obvious ataxia present with finger-to-nose or heel-to-shin testing. Biceps, brachialis, and patellar reflexes are equal bilaterally. No leg swelling or pain was noticed or reported at the time of examination. History and physical exam indicated both MRI and CT venogram of the brain with and without contrast. MRI and CT demonstrated nonocclusive mild dural venous sinus thrombosis within torcula and straight sinus, as well as nonocclusive superior sagittal sinus, rounded filling defect suggesting possible dural sinus thrombosis. There was no evidence of acute intracranial infarct or abnormal intracranial enhancement, but there was evidence of this disruption of normal flow and filling defects in superior sagittal sinus measuring up to 7 mm in size on MRI and to 8.8 mm on CT. MRI and CT imaging demonstrating a sagittal sinus filling defect in Figure 1 and Figure 2, respectively.

**Figure 3:**
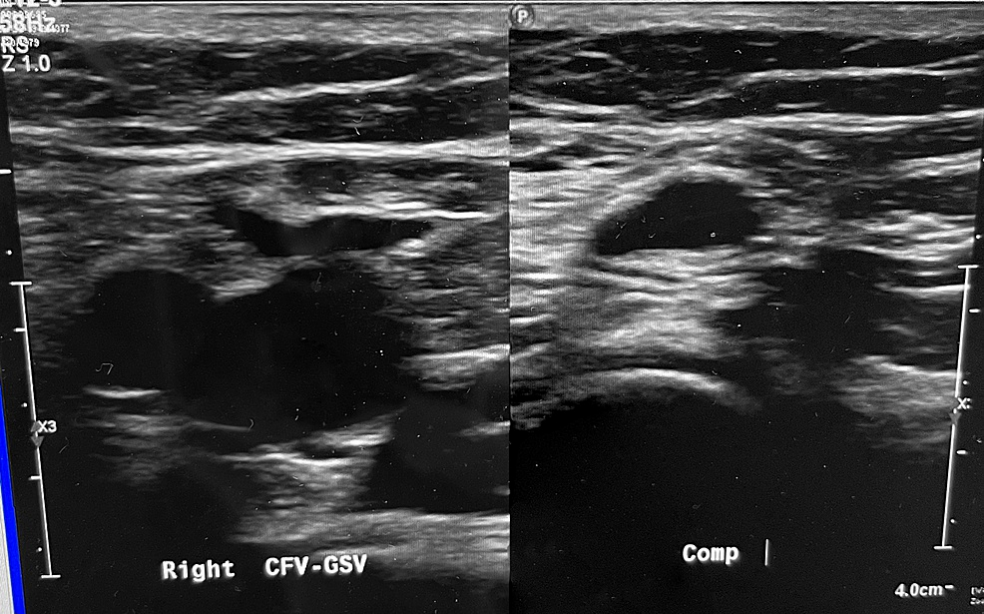
Doppler US Without (left) and With Compression (right) Suggesting Venous Thrombosis

 At this time, a diagnosis of dural venous thrombosis secondary to hypercoagulable state was made, and she was admitted to an inpatient unit. She denies any history of blood clots and leg-specific clots. For completion of her workup for other thrombotic events, ultrasound-based vascular Doppler of the bilateral lower extremity venous system was done, which found one area of occlusive thrombosis within the right calf peroneal vein distribution, as seen in Figure 3. All remaining venous systems bilaterally were patent and without evidence of a second DVT. A chest CT angiogram was done, which showed no pulmonary embolism, pleural effusion, or focal pneumonia.

Medical history is significant for Lynch syndrome with status post colon cancer diagnosed while eight months pregnant, as well as status post nonmalignant pancreatic neuroendocrine tumor (NET). Her CEA was 60.5 at the time of diagnosis, thus indicating colon cancer as normal limits are <2.5 in nonsmokers like this patient. She had T4aN0G3 disease, which falls into Stage IIB for prognostic staging. This classification indicates tumor invading through the visceral peritoneum with associated gross perforation of bowel through the tumor, no positive lymph nodes, and no metastasis [9]. This was treated with both surgery and FOLFOX chemotherapy, which includes leucovorin, 5-fluorouracil, and oxaliplatin. She reached remission and had her last treatment six months prior to this event. The patient takes the contraceptive Yaz to suppress ovulation and reduce the risk of ovarian and endometrial cancer. She is at increased risk for endometrial malignancies, gastric, duodenal, and urothelial cancers due to Lynch syndrome diagnosis [10]. Surgical history includes pancreatic neuroendocrine tumor (NET) resection and large intestine reconstruction. She does not drink alcohol and has no present or historical use of illicit drugs or tobacco products. Thus, her medical history shows possible hypercoagulability from cancer history, as well as the use of Yaz, both of which predispose her to thrombus formation.

Labs were ordered numerous times throughout her hospital stay, including a thrombotic risk panel consisting of Factor V Leiden, prothrombin gene mutation, antithrombin, protein C and S, lupus anticoagulant, anticardiolipin antibodies, and anti-glycoprotein antibodies. All results of this thrombotic risk panel were reportedly negative. Other significant findings are as follows. Leukocytosis was found at admission likely due to thrombus-related damage to venous structures, which can lead to systemic inflammatory symptoms such as leukocytosis [11]. Leukocytosis was also found again later in admission, which was expected to be reactive in nature from steroid therapy she was given later in the hospital stay. She presented with thrombocytosis at 418 x103/uL on admission as well. Research by Monreal et al. has indicated that this can often be a significant indicator for a lack of pulmonary embolism (PE) in those with venous thrombosis/thromboembolism, which was indeed true for this patient, as her thromboembolism was a dural venous sinus and leg DVT, while evidence of PE was ruled out [12]. Although high hematocrit, hemoglobin, and RBC levels are often associated with increased risk of thrombosis and thromboembolism, her hematocrit was 34.7%, just below the 36% lower limit of normal in women, hemoglobin was 11.5, again just barely below the lower limit of normal in women, and RBC of 4.26 million/mcL, which is within the normal range [13]. Thus, these particular lab findings did not demonstrate an increased risk for her chances of developing DVT. PT/INR and PTT were all found within normal limits at admission, though these values did change in accordance with medical treatments including heparin and warfarin, which would be expected to alter partial thromboplastin time (PTT) and prothrombin time (PT)/international normalized ratio (INR), respectively. By the time of discharge, INR was 1.7, PT was 19.9 seconds, and PTT was 29.2 seconds thus demonstrating findings just below the 2-3 Warfarin discharge goal, elevated, and within normal limits, respectively. Finally, a D-dimer was not ordered, due to lack of specificity for DVT, and therefore its interpretation cannot be reported.

Her thrombotic risk overall during this particular event includes her recent cancer history and use of contraceptive, Yaz. In addition to these, it is important to include the risks to which she was exposed as an endurance runner, including hemoconcentration, the potential for dehydration, muscular injury, and subsequent systemic inflammation [1]. The Wells score for DVT would be 1, which places her at moderate risk of DVT [6]. The point was scored for cancer palliation within six months, and she was declared cancer-free exactly six months prior to the event. 

Throughout hospitalization, management of the patient included Fioricet followed by fentanyl and methylprednisolone, each changed to replace the previous secondary to treatment failure for headaches, Heparin was started for anticoagulation, and Yaz was discontinued, as it is associated with increased risk of venous thrombosis. Steroids, including methylprednisolone, replaced Fentanyl after an allergic reaction, which did ultimately improve her pain symptoms. As previously mentioned, this led to brief reactive leukocytosis. Warfarin was also started, which was chosen by the hematologist/oncologist since they did not believe that there was enough evidence supporting the safety and efficacy of novel oral anticoagulants in cerebral venous thromboembolisms specifically. An IV heparin bridge was used until an appropriate INR between 2 and 3 was reached prior to discharge. As previously mentioned, INR at discharge reached 1.7. She was discharged with the plan to continue anticoagulation for a minimum of three months provided that no further thrombotic events occurred.

## Discussion

We as medical professionals are trained to suspect DVT in sedentary individuals, post-surgical patients, after long travel, overweight/obese, and with increases in age, so why is there a reportable group who directly defies these rules? Marathon/endurance athletes represent a very underreported group of people at risk for DVT. Not only do our primary scoring systems like the Well’s Score exclude them, but they also present a risk for delayed diagnosis due to the population’s nature to tend towards higher pain tolerance developed in a highly conditioned athlete who may completely write off leg pain that a sedentary person would otherwise report. As seen in this patient, leg pain was not the presenting symptom and was not reported at all throughout this patient’s hospital stay. DVT was discovered incidentally while looking for potential sources of her thrombus and/or other thrombotic events. Endurance athletes and especially marathon runners are accustomed to strenuous exercise that ultimately stimulates muscular pain during training and conditioning. This higher threshold for leg pain to be perceived as abnormal puts the population at a further disadvantage. As clinicians, it is important to have a high index of suspicion for DVT when symptoms present, as they can often be subtle or nonspecific; this typically would be very low on the differential upon hearing someone is a conditioned athlete. On the contrary though, there are many risk factors for DVT in this population, including hemoconcentration, dehydration, muscular injury, and subsequent systemic inflammation [6], and for this reason, their probability for thrombosis and thromboembolism should be appropriately studied so its incidence and prevalence can be accurately reported.

Though popular opinion views athletes as purely fit and healthy, they are still susceptible to risk factors and medical comorbidities just like any other individual. For instance, this patient was not only a marathon runner but also at one point or another in her life a cancer patient, and then survivor, a mother who experienced pregnancy, and a woman on an oral contraceptive, all of which pose their own individual hypercoagulable risk. To have DVT lower on the differential in the athletic population can be a disservice to the patient when they can and often do have just as many hypercoagulable risk factors as any other person. Additionally, as previously mentioned, athleticism also has some risk factors of its own. Virchow’s triad includes hypercoagulability, venous stasis, and vessel wall injury. Like Zadow et al. mention, dehydration and subsequent polycythemia are one example of athletic hypercoagulability, travel to races and bedrest after injury are illustrative of venous stasis, and repetitive microtrauma to the venous and arterial vasculature in athletes demonstrates vessel wall injury [14]; this means that the population can reasonably have exposure to at least one but up to all three aspects of Virchow’s triad. Interestingly, this same logic has been shown to apply to a phenomenon seen in the upper extremities. Effort thrombosis, or Paget-Schroetter syndrome, presents in gymnasts, wrestlers, swimmers, and throwing sports due to repetitive upper extremity vascular trauma causing axillary/subclavian thrombosis, again demonstrating the potential for athletes to experience thromboses [15].

Sudden cardiac death and arrhythmia in young athletes, spontaneous rupture of the spleen in athletes suffering from infectious mononucleosis, rhabdomyolysis or overuse injuries, stress fractures, and asthma are just a few examples of important and well-documented injuries and medical illnesses common in athletes. DVT, however, is still underreported in athletes and presents mainly in case studies similar to this one. Venous thromboembolism can cause significant morbidity and mortality, so with evidence for this occurrence to remain uncharted would be a disservice. We hope that by presenting this case as well as supporting research for the phenomenon, this will cause future investigation into the true incidence of thrombotic events in athletes as well as heighten the awareness of the medical field to its prevalence in this population.

## Conclusions

The index of suspicion for DVTs must be high when clinicians interact with the general population, however, it is even higher when taking care of high-performance athletes. Since these individuals do not meet the normal parameters often associated with DVTs, it is easy to overlook such diagnoses when encountered, especially since this population may associate their symptoms to their strenuous training session or may not have classic symptoms at all due to developed higher pain tolerances. Nevertheless, due to this population being exposed to numerous risk factors along their athletic journey that may increase their risk of developing venous thromboembolism, it is paramount that clinicians keep this disease on their differential. This is in order to deliver the best care to these individuals. Hopefully, by presenting this case, we are able to shed more light on this phenomenon and bring attention to the fact that this disease is possible in such unexpected populations as healthy athletes. We hope to generate reasons for DVT and other VTE in endurance athletes to be studied for accurate incidence reporting while also looking more closely into the factors that predispose them as well.
